# Supplementary material for: Experimental observation of quantum mechanical fluorine tunnelling
Source: Nat Commun. 2025 Apr 29;16:4027. doi: 10.1038/s41467-025-59008-6 (PMC12041236; doi:10.1038/s41467-025-59008-6)
Supplement: Supplementary file 1 — Supplementary Information [file 41467_2025_59008_MOESM1_ESM.pdf]

Supplementary materials for "Experimental Observation of Quantum Mechanical Fluorine Tunnelling"

Carsten Müller[1], Frederik Bader[2], Frenio Redeker[3], Lawrence Conrad[2], Helmut Beckers[1], Beate Paulus[2*], Sebastian Riedel[1†], Jean Christophe Tremblay[4‡]

[1]Institute for Chemistry and Biochemistry, Freie Universität Berlin, Fabeckstraße 34/36, D-14195 Berlin (Germany)

[2]Institute for Chemistry and Biochemistry, Freie Universität Berlin, Arnimallee 22, D-14195 Berlin (Germany)

[3]Department of Chemistry, Georgetown University, Washington, DC 20057, USA

[4]CNRS-Université de Lorraine, LPCT, 1 Bd Arago, 57070 Metz (France)

To whom correspondence should be addressed;
[*]E-mail: b.paulus@fu-berlin.de, [†]E-mail: s.riedel@fu-berlin.de
[‡]E-mail: jean-christophe.tremblay@univ-lorraine.fr

Suppl. Note 1 Computational Details

As described in the Method section of the main text, the electronic structure of $[F_5]^-$ in gas phase is calculated at the coupled cluster level of theory using single, double, and perturbative triple excitations, CCSD(T) [1, 2]. All atoms are represented using an aug-cc-pVQZ basis for the planar model and an aug-cc-pVTZ basis for the linear model [3, 4]. The dipole moment is calculated at the MP2/aug-cc-pVTZ level of theory [5]. All wave function-based electronic structure calculations were performed using the MOLPRO program package [6]. A number of stationary states are reported in Fig. 3 of the main manuscript. Their interconversions can be inferred from the potential energy cuts presented in Figs. 4 and 6. To make the potential energy surfaces more understandable, a schematic representation of the connections between the different local and global minima is shown in Fig. 1.

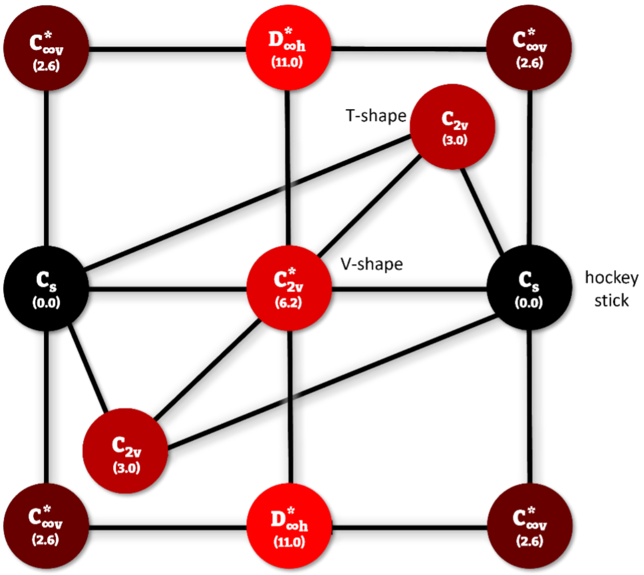

Suppl. Fig. 1 Graphical representation of how the different structures (cf. Figure 3) of $[F_5]^-$ in gas phase are connected. Transition state structures are marked with an asterisk. The numbers in parenthesis give the relative energy oft he structures in kJ/mol.

Systems with fluorine-fluorine bonds are known to show substantial correlation effects due to exceptionally strong charge shift effects in these bonds. We postulate that the linear symmetric isomer of $[F_5]^-$ should show the strongest multi-reference character of all isomers. At the CCSD(T) level of theory, the t1-diagnostic – a commonly accepted measure for multi-reference character – yields a value of 0.023. This is only very slightly above the commonly accepted threshold of 0.02, below which a system is considered single reference. In addition, CASSCF(36,20) calculations were performed for the $[F_5]^-$ transition state in $D_{\infty h}$ symmetry. The first excited state lies about 0.20 Hartree above the ground state and the leading contribution from the ground state is 0.92. We thus conclude that multi-reference character in this system is negligible and that calculations at CCSD(T) level are sufficiently accurate.

An additive guest-host potential is used to evaluate the effect of the neon atoms on the planar model. First, a model for a cavity inside the neon matrix of realistic dimension was developed by relaxing the position of a rigid $[F_5]^-$ anion inside the cavity, in order to maximize the distance to all neon atoms. This procedure was repeated for each sampling point of the potential energy surface. After relaxation, all neon atoms located at a distance smaller than the

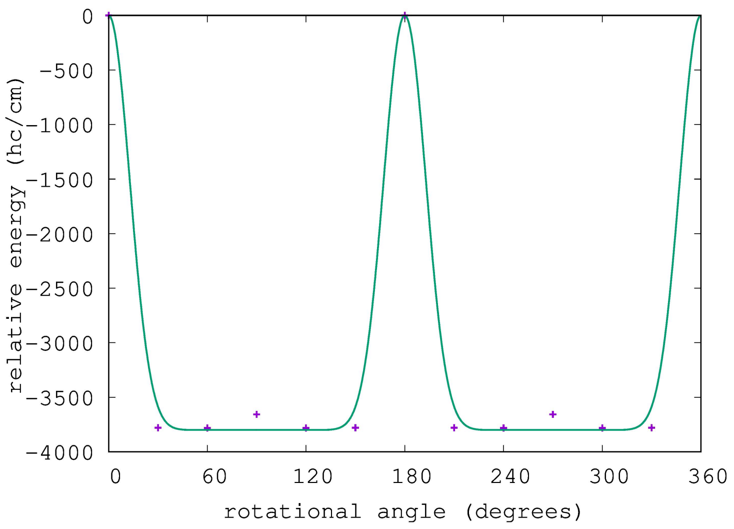

Suppl. Fig. 2 Rotational barrier for an $[F_5]^-$ rotating around its precession axis within a strongly compressed, flattened hexagonal cavity of length 10.4Å. All energies are computed at the B3LYP-D3/aug-cc-pVTZ level of theory (purple crosses) and fitted to the function $V_0 \cos^n[\varphi]$ (solid green line), where φ is the rotational angle, $n = 20$, and V_0 is the adjustment variable.

sum of van der Waals radii were removed to form a hosting cavity. An embedding energy was then calculated for each point as the difference of the MP2/aug-cc-pVTZ ground state energy for the system with and without neon matrix[5]. The corrected energies were used to adjust modified parameters of the potential energy surface. For the guest-host model used in the main text, only the potential energy surface of gas phase $[F_5]^-$ around linear configurations is considered. The interaction potential between $[F_5]^-$ and the cavity was modelled using two terminal Ne atoms. For each sampling point, an energy correction was calculated using the MP3/aug-cc-pVTZ level of theory [5]. We have checked that the MP3 relative energies are converged to within 5 meV with respect to the CCSD(T) reference values by comparing the energy difference between the potential energy minimum (498.32760697 E_h vs. -498,41890749 E_h, respectively) and the transition state structure (-498.33204190 E_h vs. -498.41465813 E_h, respectively).

For the linear model, the interaction energy of the $[F_5]^-$ anion with a neon cavity composed of the 18 closest neon atoms was computed using density functional theory (DFT) calculations [7, 8]. The Gaussian16 [9] implementation of the dispersion-corrected B3LYP-D3 functional and an aug-cc-pVTZ basis [3, 4] were used on all atoms. The rotational barriers are reported in Fig. 2 and are used to define the shape of the model potential with D_{2h} symmetry. Less compressed cavities lead to similar potential shapes but with lower rotational barriers. Bearing in mind the uncertainty on the DFT calculations, the parameters of the model used to rationalize the experimental findings are optimized for different barrier heights, ranging from 35 to 3500 cm^{-1}. The parameters used for the simulations reported in the main text can be taken from Suppl. Table 1.

Tunnelling barrier V_0 (cm^{-1})	Force constant V_1 (cm^{-1}/a_0^2)	Coupling strength V_c (cm^{-1}/a_0^2)	Moment of inertia $\mathcal{I}$ ($m_e a_0^2$)
3500	42144.065	35927.161	989.237
350	52935.299	9550.622	4287.413
35	55172.082	4398.672	13393.631

Suppl. Tab. 1 Optimized parameters for the model potential describing a linear $[F_5]^-$ anion rotating inside a Ne cavity for selected values of the tunnelling barrier V_0 in D_{2h} symmetry.

Suppl. Note 2 Spectroscopy: Planar Model

Sampling the Potential Energy Surface

For the planar model, diatom-diatom Jacobi coordinates are used (see Fig. 4 of the main manuscript for their definition). The potential energy around the global minimum is sampled uniformly in the range $R \in [2, 4]$ Å and $\theta_t \in [66°, 90°]$. Further points are sampled in the range $R \in [3, 5]$ Å and $\theta_t \in [0°, 66°]$ to cover linear configurations. All remaining coordinates are optimized under $R\theta_t$ constraints. Points at energies higher than 45 kJ/mol are discarded, amounting to 466 configurations. The dataset is symmetrized by reflection about $\theta_t = 90°$. Finally, at 17 equidistant points in $\theta_t \in [66°, 114°]$, the internal F_2 bond was sampled on five points over a range ±0.1 Å around the local optimal bond length, for 85 more configurations.

Electronic Structure Fitting

The four-dimensional potential energy function is fitted to a sum of two-body potentials for each Jacobi vector (r_1, r_2, R), which are coupled via the tunnelling coordinate, represented by the angle θ_t.

$$\begin{aligned} V(r_1, r_2, R, \theta_t) &= V_{\mathrm{F}_2}(r_1, \theta_t) + V_{\mathrm{F}_2}(r_2, \pi - \theta_t) \\ &\quad + V_{\mathrm{rep}}(R, \theta_t) + V_{\mathrm{att}}(R, \theta_t) + V_{\mathrm{coulomb}}(R, \theta_t) + V_{\mathrm{corr}}(R, \theta_t) \end{aligned} \tag{1}$$

The individual terms in these two-body potentials reflect the repulsive, ionic and dispersive characters of these interactions. For the internal vibrations along $\{r_1, r_2\}$, which remain strongly bound at all times, a modified Morse potential of the following form is chosen

$$V_{\mathrm{F}_2}(r_1, \theta_t) = D_{\mathrm{int}} \Big(1 - \exp\Big[-\alpha_{\mathrm{int}}(\theta_t)\big(r_1 - r_{e,\mathrm{int}}(\theta_t)\big)\Big]\Big)^2 \tag{2}$$

The angular dependence of the parameters reflects the smooth change in the F_2 bonding situation within the different environments, $[F_3]^- \cdots [F_2] \leftrightarrow [F_2] \cdots [F_3]^-$. Using the auxiliary variable $y = \big(\sin^2(\theta_t/2) - 1/2\big)$ to impose the correct periodicity to the parameters, the equilibrium bond length takes the form

$$r_{e,\mathrm{int}}(\theta_t) = r_{e,\mathrm{int}}^{(\mathrm{F}_3^-)} \left[\frac{1}{1 + \exp(\gamma_{r_{\mathrm{int}}} y)}\right] + r_{e,\mathrm{int}}^{(\mathrm{inf})} \left[\frac{\exp(\gamma_{r_{\mathrm{int}}} y)}{1 + \exp(\gamma_{r_{\mathrm{int}}} y)}\right] \tag{3}$$

and the spring constant function is fitted to

$$\begin{aligned} \alpha_{\mathrm{int}}(\theta_t) &= \alpha_{\mathrm{int}}^{(\mathrm{F}_3^-)} \left[\frac{1}{1 + \exp(\gamma_{\alpha_{\mathrm{int}}} y)}\right] + \alpha_{\mathrm{int}}^{(\mathrm{inf})} \left[\frac{\exp(\gamma_{\alpha_{\mathrm{int}}} y)}{1 + \exp(\gamma_{\alpha_{\mathrm{int}}} y)}\right] \\ &\quad + \alpha_{\mathrm{int}}^{(\mathrm{corr})} \cosh^{-2}(\gamma_{\alpha_{\mathrm{corr}}} y) \end{aligned} \tag{4}$$

The presence of the correction term, $\alpha_{\mathrm{int}}^{(\mathrm{corr})}$, is a consequence of the softer bond at the transition state connecting the two hockey-stick structures. The interactive plotting program Gnuplot is used to adjust simultaneously the parameters $\{r_{e,\mathrm{int}}^{(\mathrm{F}_3^-)}, r_{e,\mathrm{int}}^{(\mathrm{inf})}, \gamma_{r_{\mathrm{int}}}\}$ and $\{\alpha_{\mathrm{int}}^{(\mathrm{F}_3^-)}, \alpha_{\mathrm{int}}^{(\mathrm{inf})}, \alpha_{\mathrm{int}}^{(\mathrm{corr})}, \gamma_{\alpha_{\mathrm{int}}}, \gamma_{\alpha_{\mathrm{corr}}}\}$ to a 2D cut of the potential energy points over the whole range of values spanned by r_1 and θ_t. The values of $\{\alpha_{\mathrm{int}}^{(\mathrm{F}_3^-)}, \alpha_{\mathrm{int}}^{(\mathrm{inf})}\}$ are then manually scaled such

that the 1D Morse potential reproduces the experimental vibrational frequencies, conferring a semi-empirical character to the potential.

Parameter	Units	with embedding	w/o embedding
D_{int}	hc/(cm)	12957.0	12957.0
$\alpha_{\text{int}}^{(F_3^-)}$	Å^{-1}	1.76	1.76
$\alpha_{\text{int}}^{(\text{inf})}$	Å^{-1}	3.04	3.04
$\alpha_{\text{int}}^{(\text{corr})}$	Å^{-1}	-0.812	-0.812
$\gamma_{\alpha_{\text{corr}}}$	–	15.26	15.16
$\gamma_{\alpha_{\text{int}}}$	–	22.40	22.27
$r_{e,\text{int}}^{(F_3^-)}$	Å	1.440	1.440
$r_{e,\text{int}}^{(\text{inf})}$	Å	1.711	1.711
$\gamma_{r_{\text{int}}}$	–	27.63	27.63

Suppl. Tab. 2 Parameters of the modified Morse potential used to fit the *ab initio* data obtained at the CCSD(T)/aug-cc-pVQZ level of theory for the $[F_5]^-$ anion in planar configurations with and without embedding into a solid Ne matrix (see text).

The remaining four terms in Eq. (1) are necessary to describe the complex electronic structure of the F_2 dimer in the vicinity of the F^- anion. It is composed of a modified Lennard-Jones (6,12) potential to include the dispersive character of the $F_2 - F_2$ bond.

$$\begin{aligned} V_{\text{rep}}(R,\theta_t) &= V_r(\theta_t)\left(\frac{R_e(\theta_t)}{R}\right)^{12} \\ V_{\text{att}}(R,\theta_t) &= V_a(\theta_t)\left(\frac{R_e(\theta_t)}{R}\right)^{6} \end{aligned} \tag{5}$$

Parameter	Units	with embedding	w/o embedding
V_{r0}	hc/cm	273.895	244.8
V_{r1}	–	25.4671	24.873
V_{r2}	–	0.751998	0.7542
V_{a0}	hc/cm	-380.245	-416.93
V_{a1}	–	30.8587	34.9212
V_{a2}	–	0.869097	0.904
R_{e0}	Å	4.39405	4.4720
R_{e1}	–	2.58952	2.00399
R_{e2}	Å^{-1}	12.3056	12.318

Suppl. Tab. 3 Parameters of the Lennard-Jones potential used to fit the *ab initio* data obtained at the CCSD(T)/aug-cc-pVQZ level of theory for the $[F_5]^-$ anion in planar configurations.

A damped Coulomb potential corrects for the electrostatic character at shorter bond distances

$$V_{\text{coulomb}}(R,\theta_t) = \left(\frac{V_c(\theta_t)}{1+\exp\left[\gamma_c(\theta_t)(R-R_c(\theta_t))\right]}\right)\left(\frac{R_e(\theta_t)}{R}\right) \tag{6}$$

Finally, an attractive correction at longer distances is required to treat another dissociative channel, $[F_3]^- \cdots [F_2] \rightarrow 2F_2 + F^-$ The correction takes the following form

$$V_{\text{corr}}(R,\theta_t) = V_\Delta(\theta_t)\exp[-(R-R_\Delta(\theta_t))^2/2\sigma_\Delta^2] \tag{7}$$

Parameter	Units	with embedding	w/o embedding
V_{c0}	hc/cm	-3993.0	-3974.4
V_{c1}	hc/(cm Å)	14183.3	14173.0
V_{c2}	hc/(cm Å^2)	-20179.2	-20220.0
V_{c3}	hc/(cm Å^3)	10160.5	10098.0
R_{c0}	Å	3.16105	3.1259
R_{c1}	–	1.09635	0.9836
γ_c	Å^{-1}	0.691955	0.703433

Suppl. Tab. 4 Parameters of the damped Coulomb potential used to fit the *ab initio* data obtained at the CCSD(T)/aug-cc-pVQZ level of theory for the $[F_5]^-$ anion in planar configurations.

In Eqs. (5), (6), and (7), each parameter $P(\theta_t)$ entering the functions is represented as a symmetrized series expansion

$$P(\theta_t) = \sum_{j=0}^{n_P} P_j z^j \quad \text{with } z = \sin^2(\theta_t) \tag{8}$$

From the analysis of their parametric dependence, we found it preferable to model some terms in Eqs. (5) as exponential functions of the same symmetrized coordinate, $z = \sin^2(\theta_t)$,

$$V_r(\theta_t) = V_{r0} \exp[-V_{r1}(z - V_{r2})] \tag{9}$$

$$V_a(\theta_t) = V_{a0} \exp[-V_{a1}(z - V_{a2})] \tag{10}$$

$$V_\Delta(\theta_t) = V_{\Delta 0} \exp[-V_{\Delta 1}(z - V_{\Delta 2})^2] \tag{11}$$

The values of these parameters were obtained by numerical adjustment of the 2D *ab initio* data spanning the full range of coordinates $\{R, \theta_t\}$. All parameter values can be found in Suppl. Tables 2-5.

Parameter	Units	with embedding	w/o embedding
$V_{\Delta 0}$	hc/cm	-3857.65	-3859.7
$V_{\Delta 1}$	–	7.53162	4.2018
$V_{\Delta 2}$	–	1.27395	1.4459
$\sigma_{\Delta 0}$	Å	6.68042	12.054
$\sigma_{\Delta 1}$	–	-4.32969	-10.872
$\sigma_{\Delta 2}$	Å^{-1}	-1.8273	-0.61578
$R_{\Delta 0}$	Å	6.55595	6.7282
$R_{\Delta 1}$	–	-3.65872	-3.7425

Suppl. Tab. 5 Parameters of the correction potential used to fit the *ab initio* data obtained at the CCSD(T)/aug-cc-pVQZ level of theory for the $[F_5]^-$ anion in planar configurations.

To include the influence of the matrix on the potential energy surface, a correction was added to the electronic energy ground state energy. In Suppl. Tables 2-5, we observe that the effect of this embedding on the parameters is marginal upon refitting – only small variations are found on the terms of the modified Lennard-Jones potential, of the damped Coulomb potential, and of the internal potential. This indicates that the proposed potential form conveys most of the physics of the bonds in the $[F_5]^-$ anionic complex. On the other hand, the attractive correction to the potential is seen to be more affected by the embedding. Since the $[F_5]^-$ anion is very floppy, these small fluctuations will be shown to affect the vibrational frequencies to some extent.

Calculating Vibrational Eigenstates

Given the vibrational Hamiltonian for the planar model

$$\begin{aligned}\hat{H}_{\mathrm{4D}} = &-\frac{\hbar^2}{2\mu_{\text{F-F}}}\frac{\partial^2}{\partial r_a^2} - \frac{\hbar^2}{2\mu_{\text{F-F}}}\frac{\partial^2}{\partial r_b^2} - \frac{\hbar^2}{2\mu_{\mathrm{F_2\text{-}F_2}}}\frac{\partial^2}{\partial R^2} \\ &- \left(\frac{\hbar^2}{2\mu_{\mathrm{F_2\text{-}F_2}}R^2} + \frac{\hbar^2}{2\mu_{\mathrm{F\text{-}F_4}}r_{c,0}^2}\right)\hat{J}^2(\theta_t) + V(r_a, r_b, R, \theta_t)\end{aligned} \tag{12}$$

where the angular momentum operator is $\hat{J}^2(\theta_t) = \sin^{-1}(\theta_t)\frac{\partial}{\partial\theta_t}\sin(\theta_t)\frac{\partial}{\partial\theta_t}$. The reduced mass of fragment A-B is defined as $\mu_{\text{A-B}} = M_{\mathrm{A}}M_{\mathrm{B}}/(M_{\mathrm{A}} + M_{\mathrm{B}})$. The vibrational wave functions are represented in a tensor product basis of 1D discrete variable representation (DVR) bases

$$\psi_n(r_a, r_b, R, \theta_l) = \sum_{\alpha_a\alpha_b\beta\gamma} c^{(n)}_{\alpha_a\alpha_b\beta\gamma}\,\chi_{\alpha_a}(r_a)\chi_{\alpha_b}(r_b)\chi_\beta(R)\chi_\gamma(\theta_t) \tag{13}$$

Internal stretches $\{r_a, r_b\}$ use respectively 20 sine DVR functions [10] on the range $[1.164, 1.958]$ Å, 21 exponential DVR functions on the range $[1.587, 5.292]$ Å are used for the F_2 dimer distance R, and 101 Legendre DVR functions are used on the interval $[0, 180°]$

The low-lying eigenstates of the vibrational Hamiltonian are extracted using an in-house implementation of a coupled two-term Lanczos eigensolver with full reorthogonalization [11, 12]. An approximate spectral transform based on a finite series expansion is used to accelerate the convergence of the desired part of the spectrum [13, 14]. For the planar model in particular, 15 Chebyshev polynomials of the first kind are used to represent the spectral transformation $\exp[-a\,(\hat{H} - \sigma\hat{I})]$, with $a = 0.001$cm/hc and $\sigma = 3000$hc/cm. The lowest ~ 300 vibrational states are converged within the first 1000 Lanczos iterations.

Suppl. Note 3 Spectroscopy: Linear Model

Electronic Structure Sampling and Fitting

Energies of linear F_5^- configurations are sampled on the range $[1.3, 1.9]$ Å for the outer bonds (r_1, r_4), and on the range $[1.5, 2.5]$ Å for the inner bonds (r_2, r_3). Only the symmetry unique points on the grid are considered, amounting to 5886 configurations. The resulting potential energy function is computed employing permutationally invariant polynomials, as implemented in the monomial symmetrization approach of Bowman et al. [15] A continuous dipole moment surface along the molecular axis of linear F_5^- was constructed analogously, albeit with the dipole moment calculated at the MP2/aug-cc-pVTZ level of theory [5].

Vibrational Eigenstates

Given the vibrational Hamiltonian for the linear model

$$\begin{aligned}\hat{H}_{4\mathrm{D}} = -\frac{\hbar^2}{2\mu_{\text{F-F}}}\Bigg(&\frac{\partial^2}{\partial r_1^2} + \frac{\partial^2}{\partial r_2^2} + \frac{\partial^2}{\partial r_3^2} + \frac{\partial^2}{\partial r_4^2} \\ &- \frac{\partial^2}{\partial r_1 \partial r_2} - \frac{\partial^2}{\partial r_2 \partial r_3} - \frac{\partial^2}{\partial r_3 \partial r_4}\Bigg) + V(r_1, r_2, r_3, r_4)\end{aligned} \tag{14}$$

where $\mu_{\text{F-F}} = M_F/2$ are the reduced masses. The vibrational wave functions are represented in a tensor product basis of one-dimensional discrete variable representation (DVR) bases

$$\psi_n(r_1, r_2, r_3, r_4) = \sum_{\alpha_1\alpha_2\alpha_3\alpha_4} c^{(n)}_{\alpha_1\alpha_2\alpha_3\alpha_4}\, \chi_{\alpha_1}(r_1)\chi_{\alpha_2}(r_2)\chi_{\alpha_3}(r_3)\chi_{\alpha_4}(r_4) \tag{15}$$

The outer bonds $\{r_1, r_4\}$ use 21 sine DVR functions [10] on the range $[1.32, 1.96]$ Å and the inner bonds $\{r_2, r_3\}$ use 35 sine DVR functions on the range $[1.58, 2.54]$ Å. The low-lying eigenstates of the vibrational Hamiltonians are computed using a spectral-transform Lanczos eigensolver [11–14]. For the linear model, 15 Chebyshev polynomials of the first kind are used to represent the spectral transformation $\exp[-a\,(\hat{H} - \sigma\hat{I})]$, with $a = 0.001\mathrm{cm/hc}$ and $\sigma = 1800\,\mathrm{cm}^{-1}$. The lowest ~ 993 vibrational states are converged within the first 2000 Lanczos iterations. For the linear model, 25 Chebyshev polynomials of the first kind are used for the same transformation, with $a = 0.001\mathrm{cm/hc}$ and $\sigma = 3000\,\mathrm{cm}^{-1}$. The lowest ~ 3200 vibrational states are converged within the first 5000 Lanczos iterations.

Suppl. Note 4 Cartesian coordinates for selected structures

hockey-stick, C_{2v}, 0.0 kJ/mol

9	0.0000000000	-1.4002846927	-0.2060800192
9	0.0000000000	-0.0669558058	1.6404162454
9	0.0000000000	-0.2111727172	-1.5061319642
9	0.0000000000	0.7398863244	2.8401626305
9	0.0000000000	0.9385268913	-2.7683668925

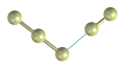

linear, $C_{\infty v}$, 2.6 kJ/mol

9	0.0000000000	0.0000000000	-1.9229636953
9	0.0000000000	0.0000000000	-3.6314968140
9	0.0000000000	0.0000000000	2.1396172486
9	0.0000000000	0.0000000000	-0.1651057592
9	0.0000000000	0.0000000000	3.5799490199

T-shape, C_{2v}, 3.0 kJ/mol

9	0.0000000000	0.0000000000	-0.0044099199
9	0.0000000000	0.0000000000	2.4655447309
9	0.0000000000	0.0000000000	3.8941171902
9	0.0000000000	-1.7395929200	0.0023739994
9	0.0000000000	1.7395929200	0.0023739994

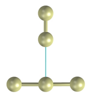

V-shape, C_{2v}, 6.2 kJ/mol

9	0.0000000000	0.0000000000	-1.2247470328
9	0.0000000000	1.5555475136	-0.1335202836
9	0.0000000000	-1.5555475136	-0.1335202836
9	0.0000000000	2.8631196490	0.7458937999
9	0.0000000000	-2.8631196490	0.7458937999

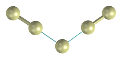

linear, $D_{\infty h}$, 11.0 kJ/mol

9	0.0000000000	0.0000000000	-0.4816120561
9	0.0000000000	0.0000000000	1.0777247058
9	0.0000000000	0.0000000000	3.0000000000
9	0.0000000000	0.0000000000	4.9222752942
9	0.0000000000	0.0000000000	6.4816120561

Suppl. Tab. 6 XYZ-coordinate files (left) and structures (right) for stationary points of $[F_5]^-$ in gas phase, as presented in Fig. 3 of the main manuscript. Energies relative to the hockey-stick structure. All distances in Å.

Linear $[F_5]^-$ in a cavity of length 10.8 Å.

9	0.000000	0.000000	3.518872
9	0.000000	0.000000	2.047337
9	0.000000	0.000000	0.000000
9	0.000000	0.000000	-2.047337
9	0.000000	0.000000	-3.518872
10	0.000000	0.000000	-5.800000
10	0.000000	0.000000	5.000000

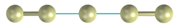

Quasi-linear $[F_5]^-$ in a cavity of length 11.4 Å.
Initial structure for optimizing the rotational barrier minimum.

9	0.221500	0.156624	0.000000
9	0.000000	0.000000	1.714747
9	0.000000	0.000000	-1.714747
9	0.000000	0.000000	3.179127
9	0.000000	0.000000	-3.179127
10	0.000000	0.000000	5.700000
10	0.000000	0.000000	-5.700000
10	0.000000	3.132483	1.566242
10	0.000000	-3.132483	1.566242
10	0.000000	3.132483	-1.566242
10	0.000000	-3.132483	-1.566242
10	2.215000	1.566242	3.132483
10	2.215000	1.566242	-3.132483
10	2.215000	-1.566242	0.000000
10	2.215000	1.566242	0.000000
10	2.215000	-1.566242	3.132483
10	2.215000	-1.566242	-3.132483
10	-2.215000	1.566242	3.132483
10	-2.215000	1.566242	-3.132483
10	-2.215000	-1.566242	0.000000
10	-2.215000	1.566242	0.000000
10	-2.215000	-1.566242	3.132483
10	-2.215000	-1.566242	-3.132483

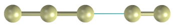

Quasi-linear $[F_5]^-$ in a cavity of length 11.4 Å.
Initial structure for optimizing the rotational barrier maximum.

9	0.200000	0.000000	0.000000
9	0.000000	0.000000	1.714747
9	0.000000	0.000000	-1.714747
9	0.000000	0.000000	3.179127
9	0.000000	0.000000	-3.179127
10	0.000000	0.000000	5.700000
10	0.000000	0.000000	-5.700000
10	0.000000	3.132483	1.566242
10	0.000000	-3.132483	1.566242
10	0.000000	3.132483	-1.566242
10	0.000000	-3.132483	-1.566242
10	2.215000	1.566242	3.132483
10	2.215000	1.566242	-3.132483
10	2.215000	-1.566242	0.000000
10	2.215000	1.566242	0.000000
10	2.215000	-1.566242	3.132483
10	2.215000	-1.566242	-3.132483
10	-2.215000	1.566242	3.132483
10	-2.215000	1.566242	-3.132483
10	-2.215000	-1.566242	0.000000
10	-2.215000	1.566242	0.000000
10	-2.215000	-1.566242	3.132483
10	-2.215000	-1.566242	-3.132483

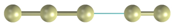

Suppl. Tab. 7 XYZ-coordinate files for representative structures of $[F_5]^-$ embedded in Ne_x. The atoms in blue define the cavity length and its orientation. Distances in Å.

Suppl. References

[1] Raghavachari, K., Trucks, G. W., Pople, J. A. & Head-Gordon, M. A fifth-order perturbation comparison of electron correlation theories. *Chem. Phys. Lett.* **157**, 479–483 (1989).

[2] Bartlett, R. J., Watts, J., Kucharski, S. & Noga, J. Non-iterative fifth-order triple and quadruple excitation energy corrections in correlated methods. *Chem. Phys. Lett.* **165**, 513–522 (1990).

[3] Dunning Jr, T. H. Gaussian basis sets for use in correlated molecular calculations. i. the atoms boron through neon and hydrogen. *J. Chem. Phys.* **90**, 1007–1023 (1989).

[4] Kendall, R. A., Dunning Jr, T. H. & Harrison, R. J. Electron affinities of the first-row atoms revisited. systematic basis sets and wave functions. *J. Chem. Phys.* **96**, 6796–6806 (1992).

[5] Møller, C. & Plesset, M. S. Note on an approximation treatment for many-electron systems. *Phys. Rev.* **46**, 618 (1934).

[6] Werner, H.-J., Knowles, P. J., Knizia, G., Manby, F. R. & Schütz, M. Molpro: a general-purpose quantum chemistry program package. *WIRE* **2**, 242–253 (2012).

[7] Hohenberg, P. & Kohn, W. Inhomogeneous electron gas. *Phys. Rev.* **136**, B864 (1964).

[8] Kohn, W. & Sham, L. J. Self-consistent equations including exchange and correlation effects. *Phys. Rev.* **140**, A1133 (1965).

[9] Frisch, M. J. *et al.* Gaussian 16 Revision C.01 (2016). Gaussian Inc. Wallingford CT.

[10] Colbert, D. T. & Miller, W. H. A novel discrete variable representation for quantum mechanical reactive scattering via the S-matrix Kohn method. *J. Chem. Phys.* **96**, 1982 (1992).

[11] Tremblay, J. C. & Carrington Jr., T. Calculating vibrational energies and wave functions of vinylidene using a contracted basis with a locally reorthogonalized coupled two-term lanczos eigensolver. *J. Chem. Phys.* **125**, 094311 (2006).

[12] Tremblay, J. C., Beyvers, S. & Saalfrank, P. Selective excitation of coupled CO vibrations on a dissipative Cu(100) surface by shaped infrared laser pulses. *J. Chem. Phys.* **128**, 194709 (2008).

[13] Tremblay, J., Füchsel, G. & Saalfrank, P. Excitation, relaxation, and quantum diffusion of CO on copper. *Phys. Rev. B* **86**, 045438 (2012).

[14] Füchsel, G., Tremblay, J. & Saalfrank, P. A six-dimensional potential energy surface for Ru(0001)(2× 2): CO. *J. Chem. Phys.* **141**, 094704 (2014).

[15] Xie, Z. & Bowman, J. M. Permutationally invariant polynomial basis for molecular energy surface fitting via monomial symmetrization. *J. Chem. Theory Comput.* **6**, 26–34 (2010).
